# Clinicopathological, epizootological, and morphological characterization of canine transmissible venereal tumor in dogs in Astana, Kazakhstan: A one-year Veterinary Diagnostic Center study

**DOI:** 10.14202/vetworld.2026.2667-2684

**Published:** 2026-06-28

**Authors:** Gulmira Kalikhanovna Murzakayeva, Assel Yerzhanovna Paritova, Karlygash Kalabayevna Ashimova, Gulzhan Omarbekova, Damir Mikdatovich Khussainov, Assylbek Zhanabayev, Yelena Bredikhina, Katira Amirova, Bulat Balabayev, Abzal Kereyev, Bibaltyn Kairgeldina

**Affiliations:** 1 Institute of Animal Science and Veterinary Medicine, S. Seifullin Kazakh Agro Technical Research University, Astana, Republic of Kazakhstan; 2 Faculty of Veterinary Medicine and Animal Engineering, Kazakh National Agrarian Research University, Almaty, Republic of Kazakhstan; 3 Department of Veterinary Medicine, A. Baitursynuly Kostanay Regional University NLC, Kostanay, Republic of Kazakhstan; 4 Institute of Veterinary and Agrotechnology, Zhangirkhan West Kazakhstan Agrotechnical University, Uralsk, Republic of Kazakhstan; 5 Veterinary Diagnostic Center “Astana”, Astana, Republic of Kazakhstan

**Keywords:** biochemistry, canine transmissible venereal tumor, cytology, dog, epidemiology, histopathology, microbiology, oncology

## Abstract

**Background and Aim:**

Canine transmissible venereal tumor (CTVT), also known as transmissible venereal sarcoma, is a naturally occurring, contagious neoplasm primarily transmitted by the transfer of viable tumor cells during mating. Although the disease is widely distributed worldwide, epidemiological and clinicopathological data from Central Asia remain scarce. This study aimed to characterize the epizootological distribution, clinical manifestations, hematological and biochemical alterations, microbiological findings, and morphological features of CTVT in dogs presented to a Veterinary Diagnostic Center in Astana, Kazakhstan.

**Materials and Methods:**

A prospective observational study was conducted from February 2024 to February 2025. A total of 2,500 dogs were screened, and 425 dogs with tumor-like lesions underwent clinical, laboratory, cytological, histopathological, and microbiological investigations. Twenty-three dogs with confirmed CTVT were included in the final analysis. Animals were categorized according to the presence (n = 13) or absence (n = 10) of purulent complications. Clinical examination, complete blood count, serum biochemical analysis, cytological evaluation using smear-imprint and fine-needle aspiration techniques, histopathological assessment, and microbiological testing were performed. Statistical analyses included Student’s t-test, Mann–Whitney U test, Fisher’s exact test, and odds ratio estimation, with significance set at p < 0.05.

**Results:**

CTVT accounted for 23 of 425 tumor-bearing dogs (5.4%). The disease was more frequently observed in males (73.9%) and in dogs aged 1.5–5 years (65.2%). Mixed-breed dogs represented the largest affected group (47.8%). Clinically, all dogs exhibited friable genital masses accompanied by hemorrhagic discharge, while purulent exudation occurred in 56.5% of cases. Extragenital involvement was identified in 8.7% of dogs. Animals with purulent complications showed significantly elevated heart rate and respiratory rate (p < 0.001), leukocytosis, reduced hemoglobin concentration, and lower hematocrit values. Biochemical analysis revealed increased concentrations of urea, creatinine, alanine aminotransferase, aspartate aminotransferase, bilirubin, and alkaline phosphatase in dogs with purulent lesions. Cytological examination demonstrated round tumor cells with eccentrically positioned nuclei, coarse chromatin, prominent nucleoli, abundant cytoplasmic vacuoles forming a characteristic “string-of-pearls” pattern, and marked mitotic activity. Histopathological findings confirmed the diagnosis and supported the observed cytomorphological features.

**Conclusion:**

This study provides the first comprehensive clinicopathological, epizootological, microbiological, and morphological characterization of CTVT in dogs from Kazakhstan. Male sex, reproductive age, and inadequate reproductive control were associated with disease occurrence. Cytoplasmic vacuolization and high mitotic activity were reliable morphological indicators of CTVT. The findings provide valuable baseline data to improve diagnosis, surveillance, and disease management strategies in Central Asia.

## INTRODUCTION

Canine transmissible venereal tumor (CTVT), also historically referred to as transmissible venereal sarcoma, is one of the most common naturally occurring neoplasms of the external genitalia in dogs worldwide, with the highest prevalence reported in regions with large populations of free-roaming and uncontrolled-breeding dogs [1, 2]. Unlike conventional neoplasms, CTVT represents a naturally occurring clonally transmissible allograft in which viable tumor cells are directly transferred between animals, most commonly during coitus and less frequently through licking, biting, or close mucosal contact [3].

The disease is characterized by the rapid growth of friable, hemorrhagic masses predominantly affecting the external genitalia, with occasional extension to adjacent tissues, including bone [3]. Although extragenital localization involving the nasal cavity, oral cavity, skin, and conjunctiva has been described, metastasis to regional lymph nodes and distant organs, including the lungs, liver, spleen, kidneys, brain, and eyes, remains uncommon, with reported rates ranging from 6.7% to 8.3% [4–7].

The epidemiology of CTVT is strongly influenced by environmental and management factors, including uncontrolled reproduction, lack of sterilization programs, and high densities of stray or semi-owned dogs [1, 2, 5]. In endemic regions of South America, Africa, and parts of Asia, the prevalence of CTVT ranges from approximately 1% to 10% of all canine tumors. In contrast, significantly lower incidence rates are observed in countries with effective population control measures, routine sterilization, and well-established veterinary infrastructure [5–7].

Modern genomic studies have confirmed that CTVT originated from a single ancient clonal lineage that has persisted for thousands of years, making it one of the oldest known continuously propagating cancer lineages in nature [8, 13–18]. This unique biological identity has positioned CTVT as an important model for investigating tumor evolution, immune evasion, and host–tumor co-adaptation. Earlier hypotheses suggesting viral involvement have been definitively replaced by evidence supporting direct cellular transmission as the primary mechanism of spread [8].

CTVT is generally classified as a round-cell tumor of mesenchymal origin and is often included among transmissible soft tissue sarcomas [8, 9]. The tumor consists of poorly differentiated round cells resembling lymphoid or plasmacytoid cells with characteristic cytoplasmic vacuolization. These features enable rapid diagnosis with minimally invasive cytological techniques such as fine-needle aspiration and imprint smears, which remain the diagnostic cornerstone in routine veterinary practice for their speed, simplicity, and cost-effectiveness [16, 17, 22–27].

Recent advances in veterinary medicine have emphasized the importance of animal models in translational research. Naturally occurring tumors such as CTVT provide valuable *in vivo* systems for studying tumor biology, immune responses, and therapeutic interventions, including comparative oncology approaches relevant to human cancer research [28]. Additionally, animal-based anatomical and pathological models play an important role in veterinary education by improving diagnostic competence and clinical understanding [29].

In parallel with these developments, artificial intelligence (AI) has emerged as a promising tool in veterinary diagnostics. AI-assisted image analysis has demonstrated potential to enhance accuracy, reproducibility, and efficiency in cytological and histopathological evaluations, although limitations remain regarding dataset quality and interpretability [30, 31]. In the context of CTVT, such approaches may support objective quantification of cellular morphology and improve diagnostic consistency in routine veterinary practice.

Astana, the capital of Kazakhstan, provides a unique setting for investigating CTVT epidemiology. Unlike many traditionally studied endemic regions with tropical or subtropical climates, Astana is characterized by a temperate continental climate and rapidly developing urban infrastructure. The city has experienced substantial growth in dog populations, including privately owned, kennel-kept, and free-roaming animals. Variability in reproductive control practices, combined with close interactions between owned and stray dogs, creates favorable conditions for disease transmission. Simultaneously, access to veterinary diagnostic services enables systematic clinical and laboratory investigation of affected animals.

Despite the global distribution and extensive investigation of CTVT in many regions of the world, information regarding its epidemiology, clinical manifestations, hematological alterations, and morphological characteristics remains extremely limited in Central Asia, particularly in Kazakhstan. Published studies from this region are scarce, and no comprehensive clinicopathological investigations have simultaneously evaluated the epizootological distribution, clinical presentation, hematological and biochemical changes, microbiological findings, and cytomorphological characteristics of CTVT in dogs. Furthermore, the influence of local environmental conditions, population management practices, and reproductive control measures on disease occurrence in urban and peri-urban canine populations remains poorly understood. This lack of region-specific evidence represents a significant knowledge gap in veterinary oncology and limits the development of effective diagnostic, surveillance, and disease management strategies for canine populations in Kazakhstan and neighboring countries.

Therefore, the present study was conducted to investigate CTVT in dogs presented to a Veterinary Diagnostic Center in Astana, Kazakhstan. By integrating epizootological, clinical, hematological, biochemical, microbiological, cytological, and histopathological assessments, the study aimed to characterize the epidemiological patterns, clinical manifestations, laboratory alterations, and morphological features of CTVT in this previously underreported region. In addition, the study sought to identify factors associated with disease occurrence and purulent complications, generate baseline regional data for future comparative investigations, and provide evidence to support improved diagnostic approaches, surveillance programs, and disease control strategies for canine populations in Central Asia.

## MATERIALS AND METHODS

### Ethical approval

The study protocol was reviewed and approved by the Ethical Commission of the Institute of Animal Science and Veterinary Medicine, S. Seifullin Kazakh Agro Technical Research University, Astana, Kazakhstan (Approval No. 1, dated March 3, 2024). The investigation was conducted in accordance with national and international guidelines governing the ethical use of animals in research and veterinary practice, including ST 33216-2014 Guidelines for the Care and Maintenance of Laboratory Animals, the International Guidelines for Biomedical Research Involving Animals, and the European Convention for the Protection of Vertebrate Animals Used for Experimental and Other Scientific Purposes. All clinical examinations, sample collection procedures, and diagnostic investigations were performed by licensed veterinarians in accordance with standard veterinary practices designed to minimize animal discomfort, stress, and the risk of injury.

The study was observational in nature and involved client-owned dogs presented to the Veterinary Diagnostic Center “Astana” for routine diagnostic evaluation and treatment. No experimental induction of disease, invasive surgical procedures beyond routine diagnostic requirements, or additional interventions unrelated to clinical management were performed. Biological samples, including tumor specimens, blood samples, and vaginal or preputial swabs, were collected only when clinically indicated as part of the diagnostic work-up. All handling and restraint procedures were conducted in accordance with accepted animal welfare standards to ensure the safety and well-being of the animals throughout the study period.

Written informed consent was obtained from all owners before the animals were enrolled in the study. Owners were informed about the purpose of the investigation, the nature of the diagnostic procedures, the intended use of collected data, and their right to withdraw their animals from the study at any stage without affecting veterinary care. All data were anonymized before analysis to protect owner confidentiality and animal identity. No animals were euthanized specifically for the purposes of this study, and all veterinary procedures complied with the euthanasia guidelines of the American Veterinary Medical Association when clinically required for unrelated medical reasons.

### Study period and location

The study was conducted from February 2024 to February 2025 at the Veterinary Diagnostic Center “Astana,” Astana, Kazakhstan. The center provides diagnostic and clinical services for a heterogeneous urban canine population comprising privately owned, kennel-kept, and free-roaming dogs. The study area represents a rapidly developing metropolitan environment in Central Asia and provides an opportunity to investigate the epidemiology and clinicopathological characteristics of CTVT under local environmental and management conditions.

### Study design

**Figure 1:**
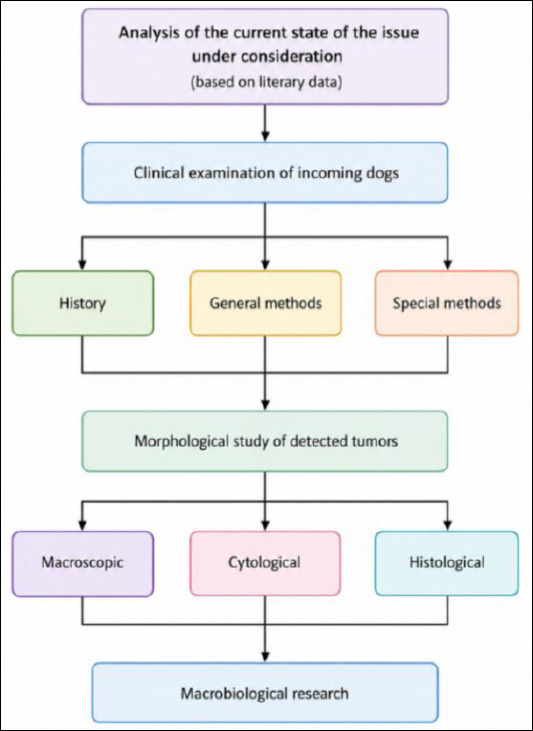
Schematic overview of the study design and diagnostic workflow used for the investigation of canine transmissible venereal tumor in dogs.

This study represents one of the largest documented case series of CTVT from Central Asia to date. A prospective observational study design was employed. During the study period, 2,500 dogs were screened, of which 425 (17.0%) presented with tumor-like lesions and underwent a standardized diagnostic evaluation that included clinical examination, cytological assessment, and histopathological examination when indicated. Among these animals, 23 dogs (5.4%) met the diagnostic criteria for confirmed CTVT and were included in the final analysis (Figure 1).

The study population consisted of dogs of different breeds, sexes, and age groups. Biological materials included samples obtained from newly diagnosed tumors, as well as vaginal and preputial swabs. To evaluate the impact of secondary infectious complications on the clinical condition of affected animals, dogs with confirmed CTVT were divided into two groups. The main group comprised 13 dogs with CTVT, accompanied by purulent genital discharge suggestive of tumor necrosis and secondary infection, whereas the control group comprised 10 dogs with CTVT without evidence of purulent exudation.

### Clinical and hematological examination

General clinical examination procedures were performed for all enrolled animals. During the collection of anamnesis, information on sex, breed, age, habitat, housing conditions, and reproductive history was recorded. Particular attention was paid to tumor growth characteristics and the occurrence of similar neoplastic lesions in mating partners and related animals. Hematological analysis was performed using a MicroCC-20Vet automated hematology analyzer (High Technology Inc., North Attleboro, MA, USA).

### Cytological and histopathological examination

**Sample collection**: Cytological samples were obtained using two complementary techniques. For the smear-imprint method, tissue sections or biopsy specimens were gently pressed onto clean glass slides to transfer cellular material. Fine-needle aspiration biopsy was performed by aspirating cells from the lesions with a sterile needle and subsequently depositing the collected material onto glass slides.

**Preparation of slides**: Immediately after collection, all samples were air-dried to preserve cellular morphology. Air-dried smears minimize cellular distortion and facilitate accurate evaluation of nuclear and cytoplasmic structures during microscopic examination.

**Staining procedure**: Cytological preparations were stained using the Pappenheim method, which consists of two sequential staining stages.

For the first stage, smears were fixed and stained using May–Grünwald stain (eosin–methylene blue) for 2–5 min. Slides were subsequently rinsed with cold water for 10–15 s to remove excess stain.

During the second stage, Romanowsky–Giemsa stain (azure–eosin) was applied using a mixture consisting of 15 mL buffer solution and 5 mL stain. The staining duration ranged from 15 to 30 min depending on sample cellularity and tissue type. After staining, slides were rinsed with cold water for 20 s and air-dried before microscopic examination.

**Microscopic examination and diagnostic confirmation**: Microscopic examination was performed using a Hospitex Micro Screen light microscope (Hospitex Diagnostics, Florence, Italy). Cytological evaluation was conducted using established veterinary pathology references, cytology atlases, and histopathology guidelines.

A definitive cytological diagnosis was established only after comprehensive evaluation of stained preparations. All laboratory procedures were performed according to standardized protocols to ensure reproducibility and diagnostic accuracy. The types and volumes of diagnostic procedures performed during the study are presented in Table 1.

**Table 1:** Types and volume of clinical, laboratory, morphological, and microbiological investigations performed during the diagnosis and characterization of canine transmissible venereal tumor in dogs enrolled in the study.

Type of work	Research material	Number of objects	Number of samples
Clinical examination – General	Dogs	425	23
Clinical examination – Laboratory	Blood samples	23	23
Morphological examination – Macroscopic	Dogs	23	-
Morphological examination – Cytological	Tumor-derived samples	23	23
Morphological examination – Histological	Tumor-derived samples	23	23

### Microbiological analysis

Clinical samples were collected under aseptic conditions. Isolation and identification of microorganisms were performed using conventional microbiological methods, including culture on selective media and microscopic examination. Antimicrobial susceptibility testing was conducted according to the methodology described by Chaikovskaya *et al*. [32]. Interpretation of antimicrobial susceptibility results was based on established clinical breakpoints. Quality-control strains were included throughout the study to ensure analytical reliability and accuracy.

### Imaging and documentation

Microphotographs were obtained using a 3.1-megapixel USB digital eyepiece camera (Altami UCMOS03100KPA-U-NA-NC-SQ-BA, Altami, Saint Petersburg, Russia). Macrophotographs were captured using Canon PowerShot A1200 camera (Canon Inc., Tokyo, Japan) and Nikon D5100 Kit (Nikon Corporation, Tokyo, Japan) digital cameras.

### Statistical analysis

Statistical analysis was performed using IBM SPSS Statistics version 27.0.1 (IBM Corp., Armonk, NY, USA). The distribution of continuous variables, including body temperature, heart rate, and respiratory rate, was evaluated using the one-sample Kolmogorov–Smirnov test.

Normally distributed variables, including body temperature and heart rate, were analyzed using the independent-samples Student’s t-test and are presented as mean ± standard error. Variables with non-normal distributions, including respiratory rate and age, were analyzed using the Mann–Whitney U test and are presented as median with interquartile range.

Categorical variables, including sex and breed status, were analyzed using contingency tables and Fisher’s exact test. The risk of developing complications was assessed by calculating odds ratios with corresponding 95% confidence intervals. Statistical significance was established at p < 0.05.

### Methodological significance

This study is among the few investigations conducted in Central Asia that integrates clinical examination, hematology, biochemistry, cytology, histopathology, and microbiology within a single diagnostic framework. The comprehensive multidisciplinary approach adopted in the present study provides a detailed characterization of CTVT in a previously underreported geographical region and contributes valuable baseline data for future epidemiological and clinicopathological investigations.

## RESULTS

### Occurrence of CTVT

**Figure 2:**
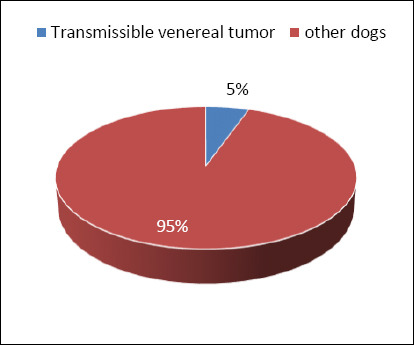
Percentage of dogs diagnosed with canine transmissible venereal tumor (%, n = 23).

Out of 2,500 examined dogs, 425 (17.0%) presented with tumor-like lesions. Among these, 23 cases were confirmed as CTVT, accounting for 5.4% of all neoplasms diagnosed during the study period (Figure 2). This proportion represents one of the first documented epidemiological benchmarks for CTVT in Kazakhstan, where previously published data are lacking. Analysis of the collected anamnestic data revealed a clear association between the development of CTVT and several factors, including sex, age, and breed of the animals, as well as housing conditions and the degree of mating control. CTVT was diagnosed more frequently in males (73.9%) than in females (26.1%), with the highest prevalence in dogs aged 1.5–5 years (65.2%). Males accounted for 73.9% of cases, in contrast to some reports from tropical regions where females may predominate, likely due to differences in mating dynamics, population structure, and animal management practices.

**Figure 3:**
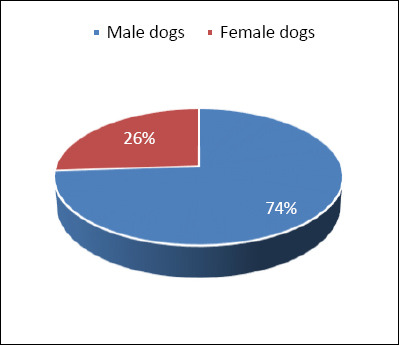
Incidence of canine transmissible venereal tumor according to sex of animals (%, n = 23).

CTVT was most frequently diagnosed in male dogs (17 cases, 73.9%), whereas only six cases (26.1%) were recorded in female dogs (Figure 3).

**Figure 4:**
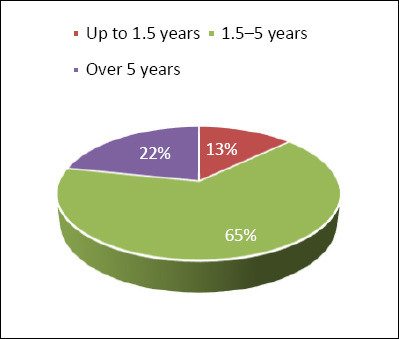
Incidence of canine transmissible venereal tumor according to age of animals (%, n = 23).

The age-related susceptibility of dogs to the disease is presented in Figure 4. The highest incidence was observed in dogs aged 1.5–5 years, with 15 affected animals (65.2%). The lowest incidence was recorded in dogs younger than 1.5 years, accounting for three cases (13.0%). In dogs older than 5 years, the disease was diagnosed in five animals (21.8%).

**Figure 5:**
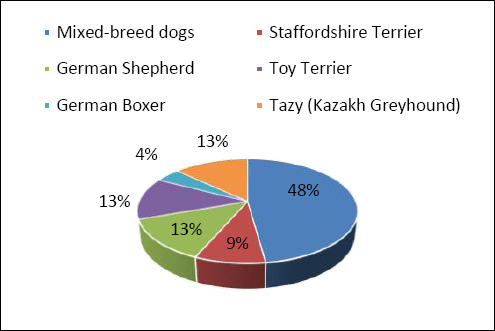
Genetic predisposition to canine transmissible venereal tumor (%, n = 23).

Breed-related predisposition to CTVT is shown in Figure 5. Among the affected animals, mixed-breed dogs were most frequently diagnosed, accounting for 11 cases (47.8%). Three cases each were recorded in German Shepherds, Toy Terriers, and Greyhounds (13.0% per breed). Staffordshire Bull Terriers accounted for two cases (8.7%), while a single case was identified in a German Boxer (4.3%) of the total number of affected dogs.

Analysis of the anamnestic data revealed that CTVT was most frequently diagnosed in intact male dogs aged 1.5–5 years that were kept in the private housing sector.

At the Astana Veterinary Diagnostic Center, of the 2,500 dogs examined during the study period, oncological diseases were diagnosed in 425 (17.0%). Among these cases, 23 dogs were diagnosed with CTVT, accounting for 5.4% of all oncological pathologies. Of the 23 affected dogs, CTVT was more common in males (17 cases, 73.9%) than in females. With respect to age, the highest incidence was observed in dogs aged 1.5–5 years, comprising 15 cases (65.2%).

Regarding breed distribution, CTVT was most frequently diagnosed in mixed-breed dogs (11 cases, 47.8%), with the remaining cases in purebred dogs (Figure 5).

### Clinical signs and tumor localization

**Figure 6:**
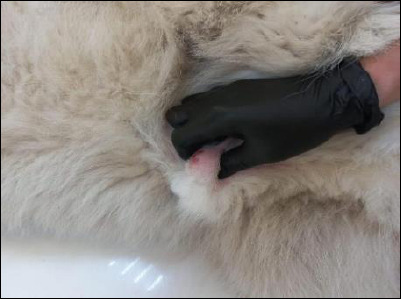
A purebred male dog aged 4 years with canine transmissible venereal tumor located in the bulb of the penis and bloody discharge.

Clinical signs suggestive of CTVT were identified in all 23 affected animals. The most characteristic manifestations included bloody discharge from the external genitalia and the presence of tumor-like masses on the penis, preputial mucosa, or vaginal mucosa (Figure 6).

**Figure 7:**
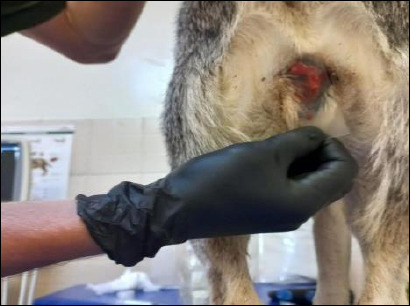
A purebred female dog aged 3 years and 7 months with canine transmissible venereal tumor complicated by purulent discharge.

In some cases, tumor growth was accompanied by abundant genital discharge mixed with blood, yellowish-green in color, and characterized by an unpleasant odor (Figure 7).

All 23 dogs presented with genital tumors characterized by friable, bleeding masses located on the penis, prepuce, or vaginal mucosa. All affected dogs exhibited characteristic genital tumors presenting as friable, highly vascular, and easily bleeding masses located in the prepuce, penis, or vaginal mucosa.

**Figure 8:**
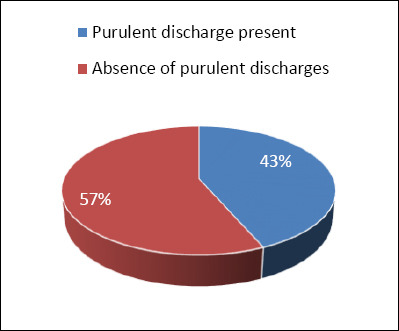
Presence or absence of purulent discharge (%; n = 23).

Purulent discharge was observed in 13 of 23 dogs (56.5%) (Figure 8), consistently calculated using the total confirmed cohort. The high rate of secondary purulent complications (56.5%) in a temperate Central Asian setting represents a clinically relevant finding, as most published data originate from tropical and subtropical regions.

Dogs with purulent discharge showed more pronounced systemic clinical signs, including lethargy, reduced appetite, and decreased responsiveness. No alternative denominators or subgroup-based percentages were used to maintain statistical consistency across the study. Extragenital localization was observed in two of 23 cases (8.7%), involving the conjunctiva and forelimb.

**Figure 9:**
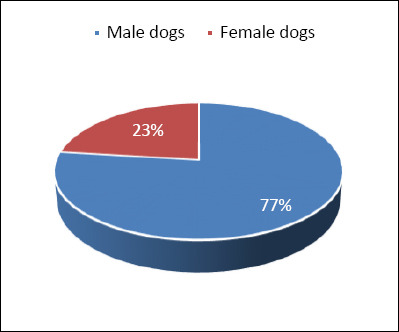
Purulent discharge according to sex of dogs (%, n = 13).

Purulent discharge was detected in 13 dogs, including 10 males (76.92%) and three females (23.07%) (Figure 9).

### Physiological indicators

In animals without purulent discharge, physiological parameters were normal (Table 2). In some cases, decreased appetite and activity were observed. The analysis of physiological parameters in dogs without purulent discharge (n = 10) demonstrated that all measured indicators remained within established reference ranges, indicating a clinically stable condition of the animals.

**Table 2:** Physiological indicators of dogs in the absence of purulent discharge (M ± m, n = 10).

No.	Temperature reference range (°C)	Temperature (°C)	Heart rate reference range (beats/min)	Heart rate (beats/min)	Respiratory rate reference range (breaths/min)	Respiratory rate (breaths/min)
1	37.5–39.5	37.7	70–100	71	10–18	12
2	37.5–39.5	38.0	70–100	80	10–18	16
3	37.5–39.5	37.5	70–100	86	10–18	18
4	37.5–39.5	39.2	70–100	95	10–18	11
5	37.5–39.5	38.6	70–100	90	10–18	13
6	37.5–39.5	37.7	70–100	81	10–18	12
7	37.5–39.5	38.2	70–100	75	10–18	15
8	37.5–39.5	39.1	70–100	96	10–18	14
9	37.5–39.5	37.9	70–100	80	10–18	17
10	37.5–39.5	38.1	70–100	86	10–18	16
M ± m	-	38.17 ± 0.25	-	84 ± 6.3	-	14.4 ± 1.1

The mean body temperature was 38.17°C ± 0.25°C, with individual values ranging from 37.5°C to 39.2°C, which fully corresponds to the physiological norm for the species (37.5°C–39.5°C). The absence of hypo- or hyperthermia suggests no systemic inflammatory response or impairment of thermoregulation.

Heart rate ranged from 71 to 96 beats/min, with a mean value of 84 ± 6.3 beats/min, which falls within the reference interval (70–100 beats/min). These findings indicate the absence of tachycardia or bradycardia and suggest a compensated and physiologically adequate functional state of the cardiovascular system.

Respiratory rate ranged from 11 to 18 breaths/min, with a mean value of 14.4 ± 1.1 breaths/min, which is consistent with normal physiological values (10–18 breaths/min). The absence of tachypnea or bradypnea indicates preserved pulmonary ventilation function and no evidence of respiratory insufficiency.

Thus, the overall results indicate no clinically significant deviations in the evaluated physiological parameters. This group of animals may therefore be appropriately used as a control group in comparative studies aimed at assessing pathological conditions associated with inflammatory and intoxication processes.

Analysis of physiological parameters demonstrated that the mean body temperature in both groups (38.61 ± 0.70°C and 38.20 ± 0.59°C) remained within the reference range (37.5°C–39.5°C), with no statistically significant difference between them (p = 0.152) (Table 3).

**Table 3:** Physiological indicators of dogs in the presence of purulent discharge (M ± m, n = 13).

No.	Temperature reference range (°C)	Temperature (°C)	Heart rate reference range (beats/min)	Heart rate (beats/min)	Respiratory rate reference range (breaths/min)	Respiratory rate (breaths/min)
1	37.5–39.5	39.3	70–100	101	10–18	30
2	37.5–39.5	38.0	70–100	98	10–18	31
3	37.5–39.5	39.1	70–100	96	10–18	38
4	37.5–39.5	37.8	70–100	105	10–18	39
5	37.5–39.5	39.5	70–100	99	10–18	33
6	37.5–39.5	38.3	70–100	106	10–18	30
7	37.5–39.5	37.9	70–100	105	10–18	38
8	37.5–39.5	39.0	70–100	106	10–18	37
9	37.5–39.5	38.5	70–100	108	10–18	30
10	37.5–39.5	38.1	70–100	107	10–18	40
11	37.5–39.5	39.3	70–100	97	10–18	39
12	37.5–39.5	37.6	70–100	102	10–18	31
13	37.5–39.5	39.5	70–100	98	10–18	36
M ± m	-	38.6 ± 0.19	-	102.2 ± 1.2	-	34.8 ± 1.1

**Table 4:** Degree of manifestation of clinical symptoms (n = 23).

Clinical signs	Absence of purulent discharge (n = 10)	Presence of purulent discharge (n = 13)
Anxiety	–/+	++
Lack of appetite	–/+	++
Decreased physical movement	–	++

++ = moderately visible symptom, + = weakly visible sign, – = sign not observed.

However, dogs in the main group with purulent discharge exhibited a pronounced cardiovascular and respiratory response. The mean heart rate in dogs with purulent discharge was 102.15 ± 4.22 beats/min, exceeding the upper reference limit (70–100 beats/min), whereas in the control group, it remained within the normal range (84.00 ± 8.17 beats/min, p < 0.001).

The most marked differences were observed in respiratory rate. In the main group, the median respiratory rate was 36 breaths/min, significantly higher than the reference range (10–18 breaths/min), while in the non-purulent group, it remained within normal limits (14.50 breaths/min, p < 0.001).

These findings indicate that purulent tumor breakdown in CTVT is associated with a systemic physiological response characterized by tachycardia and tachypnea, even in the absence of a significant febrile reaction.

**Figure 10:**
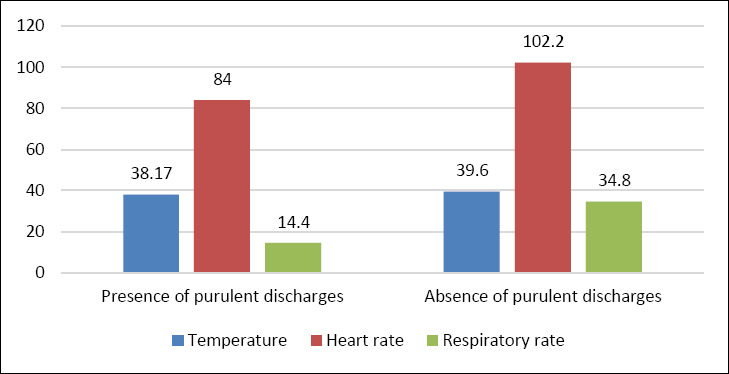
Mean values of physiological indicators (n = 23).

Figure 10 shows the differences in physiological indicators of dogs with complicated and uncomplicated courses of the tumor process.

Transmissible venereal tumor was diagnosed in 23 dogs, which were divided into two groups according to clinical signs: purulent and non-purulent forms (Tables 5 and 6). Based on physiological parameters, including body temperature, respiratory rate, and heart rate, dogs with purulent inflammation had higher values than those without purulent inflammation.

**Table 5:** Comparative demographic characteristics and risk analysis between groups (n = 23).

Indicator/category	Group with purulent process (n = 13)	Group without purulent process (n = 10)	Significance (p)	Odds ratio (95% Confidence interval)
Sex (male/female)	10/3	7/3	1.000^1^	1.429 (0.220–9.262)
Age (years), M ± SD	3.83 ± 1.92	3.27 ± 2.14	0.420^2^	-
Breed status (yes/no)	5/8	6/4	0.414^1^	0.417 (0.077–2.253)

1 Fisher’s exact test,

2 Mann–Whitney U test.

**Table 6:** Physiological parameters for various forms of canine transmissible venereal tumor.

Indicator	Reference range	Group with purulent discharge (n = 13)	Group without purulent discharge (n = 10)	Significance (p)
Body temperature (°C)	37.5–39.5	38.61 ± 0.70	38.20 ± 0.59	0.152^1^
Heart rate (beats/min)	70–100	102.15 ± 4.22	84.00 ± 8.17	<0.001^1^
Respiratory rate (breaths/min)	10–18	36.00 (8.0)*	14.50 (4.0)*	<0.001^2^

* Data are presented as median (interquartile range),

1 Student’s t-test,

2 Mann–Whitney U test.

According to the severity of clinical manifestations, in the absence of purulent discharge, 10 dogs exhibited mild anxiety, decreased appetite, and reduced physical activity. In contrast, in the presence of purulent discharge, 13 dogs showed pronounced anxiety, loss of appetite, and markedly reduced physical activity.

### Hematological indicators of CTVT

The physiological parameters of dogs with a tumor process accompanied by purulent discharge differed from those observed in the previous group (Table 7). These animals exhibited a moderate increase in body temperature (38.6 ± 0.19°C) and heart rate (102.2 ± 1.2 beats/min), as well as a marked increase in respiratory rate (34.8 ± 1.1 breaths/min).

**Table 7:** Results of complete blood count (M ± m, n = 23).

Indicator	Reference values	Dogs without purulent discharge (n = 10)	Dogs with purulent discharge (n = 13)
Hematocrit (%)	32.5–41.5	26.0 ± 1.1	24.0 ± 1.3
Hemoglobin (g/dL)	11.0–14.5	9.5 ± 0.25	9.01 ± 0.27
Erythrocyte count (million/μL)	3.50–4.70	3.0 ± 0.2	2.9 ± 0.2
Leukocyte count (×10³/μL)	4.5–10.0	21.0 ± 0.4	26.0 ± 0.6
Platelets (×10³/μL)	160–380	220 ± 15	198 ± 12

The severity of the symptoms is shown in Table 4.

Clinically, the affected dogs appeared lethargic, demonstrated reduced appetite, and showed delayed responses to external stimuli.

**Figure 11:**
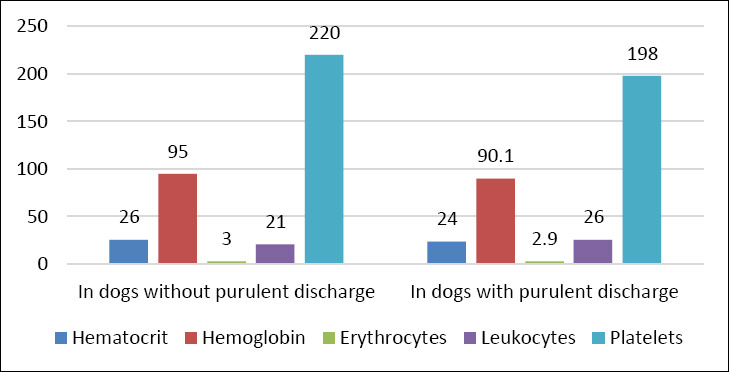
Mean values of complete blood count (n = 23).

The relationships among the studied indicators at different stages of the tumor process are shown in Figure 11. The analysis showed that dogs with purulent discharge had elevated reference values for many parameters. In particular, it was observed that the levels of urea, creatinine, alanine aminotransferase, aspartate aminotransferase, bilirubin, and alkaline phosphatase increased slightly.

**Figure 12:**
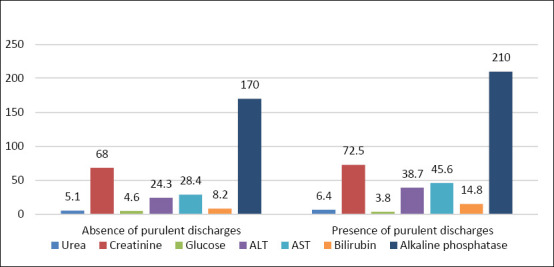
Average values of biochemical blood tests (n = 23).

However, the glucose reference values were slightly reduced in dogs with purulent discharge compared to dogs without purulent discharge (Table 8 and Figure 12).

**Table 8:** Results of biochemical blood tests (M ± m, n = 23).

No.	Indicator	Reference values	Dogs without purulent discharge (n = 10)	Dogs with purulent discharge (n = 13)
1	Urea	2.50–6.00	5.1 ± 0.3	6.4 ± 0.4
2	Creatinine	44.00–88.00	68.0 ± 2.1	72.5 ± 3.0
3	Glucose	3.33–5.55	4.6 ± 0.2	3.8 ± 0.3
4	Alanine aminotransferase	0.00–39.00	24.3 ± 1.7	38.7 ± 2.1
5	Aspartate aminotransferase	0.00–51.00	28.4 ± 2.1	45.6 ± 2.5
6	Total bilirubin	<17.00	8.2 ± 0.5	14.8 ± 0.7
7	Alkaline phosphatase	189 ± 15.47	170 ± 12	210 ± 14

Based on hematological parameters and biochemical blood analysis, the studied dogs with purulent and non-purulent inflammation had decreased hemoglobin and hematocrit values, as well as reduced erythrocyte counts, due to blood loss. The number of leukocytes increased 4–5 times due to the inflammatory process. In biochemical studies, alkaline phosphatase was elevated in purulent inflammation, whereas it was normal in non-purulent inflammation. In sick dogs, aspartate aminotransferase and alanine aminotransferase levels were elevated due to impaired liver cell function.

### Macroscopic characteristics of CTVT

Pale pink, dark pink, or dark red, small, easily eroded, and profusely bleeding lesions were found on the foreskin or vaginal mucosa. These lesions were usually located on a broad base. Their diameter varied considerably from 1.5 to 11 cm.

In female dogs, the tumor was most often located in the mucous membrane of the vaginal opening (Figure 15). In male dogs, the tumor was found in the glans penis, penile head, or prepuce.

CTVT, located in the mucous membrane of the vaginal opening, had a dense structure. CTVT, located in the glans penis, had a soft structure and bled easily. CTVT located in the mucous membrane of the foreskin had a cluster-of-grapes appearance and bled easily.

In most cases (20 dogs, 87.0%), the tumor was soft in consistency, and its parts were easily separated without surgical instruments. In three dogs (13.0%), the tumor was observed as a dense structure (Figure 14).

**Figure 13:**
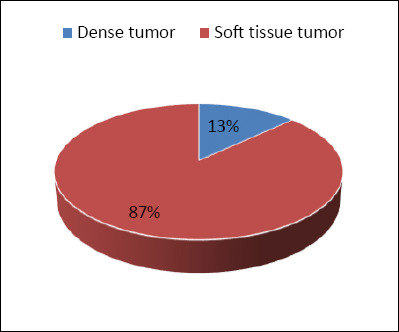
Proportion of tumors with different structures (%, n = 23).

In two cases, extragenital tumors were detected: one was located in the forelimb of the dog, and the other was located in the conjunctiva. The ratio of CTVT localization types is shown in Figure 15.

**Figure 14:**
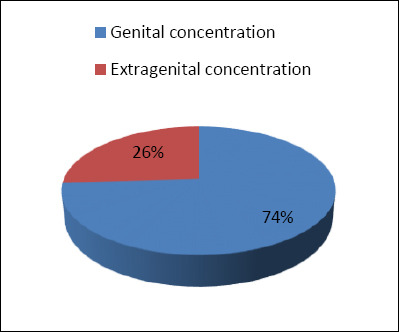
Proportion of different types of CTVT placement (%, n = 23).

Macroscopically, we divided CTVT in dogs into two groups based on consistency: dense tumors (13%) and loose tumors (87%), which were observed in advanced cases. Genital lesions were present in 17 dogs (74%), and extragenital lesions were present in six dogs, which was 26%.

### Microscopic characteristics of CTVT

**Figure 15:**
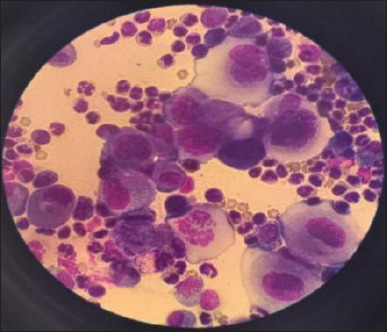
Canine transmissible venereal tumor cytological preparation. A smear was taken from the superficial layer of the tumor. Large round cells with prominent nuclei, medium-sized light-blue cytoplasm, and punctate vacuoles are visible. Pappenheim staining, 1000× magnification.

For cytological examination, smears were obtained from tumor tissue. The collected smears were air-dried and subsequently stained using the Pappenheim method (Figure 15).

The stained preparations were examined under a light microscope. Microscopic analysis revealed round-shaped cells with large, eccentrically located nuclei, dense nuclear chromatin, and clearly visible single or multiple nucleoli. The cytoplasm was stained light blue and present in moderate amounts. Characteristic cytoplasmic vacuolization in the form of a “pearl chain,” as well as high mitotic activity, was observed.

**Figure 16:**
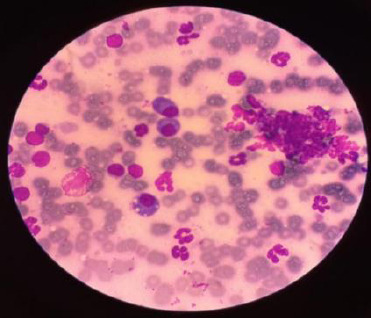
Canine transmissible venereal tumor. A smear was taken from the surface of the tumor. Numerous small vacuoles are observed. Pappenheim staining, 400× magnification.

One of the characteristic features of CTVT is the presence of numerous fine punctate, pearl-like cytoplasmic vacuoles (Figure 16). In addition, pronounced mitotic activity was noted. Against this background, an increased number of plasma cells, lymphocytes, macrophages, and neutrophils was detected.

Figure 16 presents the histological appearance of biopsy material obtained from a tumor of extragenital localization, based on which the diagnosis of CTVT was confirmed.

### Microbiological findings

Microbiological analysis of tumor-associated samples did not reveal consistent or clinically significant bacterial growth in most cases. In several samples with purulent discharge, bacterial colonies were detected; however, these were identified as opportunistic contaminants of low diagnostic significance.

Due to the inconsistent nature and limited reproducibility of microbial isolation across samples, microbiological findings were not included as primary outcome variables in the statistical analysis. The results suggest that purulent discharge observed in a proportion of cases is more likely associated with tumor necrosis and secondary contamination rather than a specific infectious agent.

## DISCUSSION

### Regional significance and study scope

It should be noted that the present study did not evaluate treatment strategies for CTVT. The focus was restricted to diagnostic assessment using clinical, cytological, and histopathological methods. Therapeutic outcomes were beyond the scope of this investigation.

This study represents the first comprehensive clinicopathological characterization of CTVT in Kazakhstan and one of the very few reports from temperate Central Asia. Previous global analyses have highlighted a marked lack of data from this region, despite the widespread presence of disease in comparable epidemiological settings. Therefore, the present findings address a significant geographic gap and provide baseline data for future surveillance and control strategies.

Unlike most published studies originating from tropical and subtropical regions, the current work reflects CTVT dynamics in a temperate urban environment, where dogs are predominantly privately owned but often experience incomplete reproductive control. This context offers a distinct epidemiological model that differs from classical stray-dominated transmission systems.

### Occurrence of CTVT in Astana

The prevalence of CTVT observed in this study (5.4% of all neoplasms) is consistent with reports from endemic regions worldwide. Studies from South America, Africa, and Asia have documented prevalence rates ranging from approximately 1% to 10% of canine tumors, particularly in areas with large free-roaming dog populations and limited reproductive control [36, 37]. In contrast, significantly lower prevalence has been reported in countries with effective sterilization programs and controlled breeding [5]. Therefore, the findings from Astana reflect epidemiological patterns typical of regions with active stray dog populations and uncontrolled reproduction dynamics.

### Sex, age, and breed-related patterns

The predominance of male dogs (73.9%) observed in this study is consistent with several reports but differs from others showing either balanced or female-predominant distributions [5, 36]. This variation is more likely explained by behavioral and environmental factors rather than biological susceptibility.

Male dogs generally demonstrate higher roaming activity, increased territorial behavior, and more frequent mating attempts, which significantly elevate exposure risk [36]. Additionally, clinical detection bias may contribute, as genital lesions in males are often more visible and prompt earlier veterinary consultation. Thus, sex differences should be interpreted primarily as differences in exposure rather than intrinsic predisposition [10].

Mixed-breed dogs constituted the largest proportion of affected animals in the present study. However, this finding should be interpreted cautiously, as breed distribution reflects the underlying population structure in Astana rather than true genetic susceptibility.

As emphasized by Das and Das [5] and Mukaratirwa and Gruys [11], apparent breed predisposition in CTVT is strongly influenced by ownership patterns, roaming behavior, and access to veterinary care. Therefore, CTVT should be considered a behavior-associated and population-dependent disease rather than a breed-related condition.

The age distribution observed in the present study, with the highest frequency occurring in dogs aged 1.5–5 years, is consistent with the period of peak sexual activity and reproductive behavior. Similar observations have been reported in previous epidemiological studies, where sexually mature dogs exhibited the highest risk of tumor transmission due to increased opportunities for direct contact during mating [5, 10, 36].

### Clinical presentation and purulent complications

Clinically, CTVT presented as friable, highly vascular genital masses accompanied by bloody discharge, consistent with classical descriptions [35]. The presence of purulent discharge in 56.5% of cases was associated with tumor ulceration and secondary bacterial infection, a complication commonly reported in advanced disease stages [27].

Dogs with purulent discharge exhibited more pronounced systemic clinical signs, including lethargy, reduced appetite, tachycardia, and tachypnea. These findings indicate that secondary inflammatory processes significantly influence the clinical severity of the disease and contribute to the deterioration of the general condition of affected animals.

The absence of severe systemic temperature elevation in many cases further supports a subacute-to-chronic inflammatory process rather than acute systemic sepsis.

### Hematological and biochemical alterations

The hematological and biochemical changes observed in this study should be interpreted as secondary effects of tumor progression and infection rather than primary tumor-driven metabolic alterations.

Leukocytosis most likely reflects a systemic inflammatory response associated with secondary bacterial infection. Anemia is consistent with chronic blood loss from friable tumor tissue. Elevated alanine aminotransferase and aspartate aminotransferase activities, together with increased bilirubin concentrations, may indicate systemic inflammatory stress or mild hepatocellular dysfunction secondary to infection and toxemia rather than direct neoplastic infiltration [37, 38].

The observed increase in alkaline phosphatase activity in dogs with purulent inflammation further supports the presence of systemic inflammatory responses and tissue damage. Similarly, the slight reduction in glucose concentrations in affected dogs may reflect reduced appetite and altered metabolic status associated with chronic disease.

Overall, the hematological and biochemical findings suggest that advanced CTVT accompanied by purulent complications can induce measurable systemic physiological disturbances, despite the predominantly localized nature of the tumor.

### Macroscopic and microscopic characteristics

Tumors were predominantly soft, friable, and highly vascular, ranging from 1.5 to 11 cm in diameter, consistent with previous descriptions by Stockmann *et al*. [10]. These features reflect the loose stromal architecture and high cellular density characteristic of CTVT.

Macroscopically, most tumors exhibited a grape-like appearance and were prone to bleeding upon manipulation. The predominance of soft tumors (87.0%) observed in the present study agrees with previous reports describing advanced-stage lesions characterized by extensive vascularization and tissue fragility.

The cytological characteristics observed in this study, including large, round cells; a high nuclear-to-cytoplasmic ratio; eccentrically located nuclei; coarse chromatin; prominent nucleoli; and frequent mitotic figures, are fully consistent with established descriptions of CTVT [5, 10].

One of the most notable microscopic findings was the presence of characteristic cytoplasmic vacuolization arranged in a “string-of-pearls” pattern. In addition, marked mitotic activity and inflammatory cell infiltrates, including plasma cells, lymphocytes, macrophages, and neutrophils, were consistently observed.

### Diagnostic significance of cytology and histopathology

A particularly important diagnostic feature identified in this study was the presence of cytoplasmic vacuolization forming a characteristic “string-of-pearls” pattern. This feature is widely recognized as a highly specific cytomorphological marker of CTVT and has been emphasized by Meuten [27] and Cowell *et al*. [35] as one of the most reliable cytological criteria for rapid diagnosis.

The findings of the present study further support the value of cytology as a rapid, minimally invasive, and cost-effective diagnostic tool for routine veterinary practice. Cytological examination provided sufficient information for diagnosis in most cases and was particularly useful for initial clinical assessment.

Histopathological findings confirmed cytological results, particularly in extragenital cases, where tissue architecture was essential for definitive diagnosis. Similar observations have been reported by Mukaratirwa and Gruys [11], who highlighted the importance of histological confirmation for the localization of atypical tumors.

Overall, the diagnostic value of cytology, particularly the characteristic “string-of-pearls” vacuolization pattern, remains central to the rapid and reliable identification of CTVT, as supported by Cowell *et al*. [35] and Meuten [27].

### Extragenital localization

Extragenital localization was observed in 8.7% of cases. Although slightly higher than commonly reported values (<10%), this remains within the expected biological variation and is likely attributable to autoinoculation via licking, biting, or traumatic implantation in environments with high animal density [36].

The occurrence of tumors in the conjunctiva and forelimb supports previous observations that CTVT is not exclusively restricted to the genital tract and may develop in non-genital sites following direct implantation of viable tumor cells. These findings emphasize the importance of considering CTVT in the differential diagnosis of atypical masses occurring outside the reproductive system.

Overall, the cytological, clinical, and epidemiological features observed in this study are in agreement with the global literature. CTVT continues to be recognized as a transmissible clonal allograft tumor with distinct morphological features and strong dependence on population dynamics rather than genetic factors [8, 13–18].

### Study limitations

This study has several limitations that should be acknowledged. First, the relatively small sample size (n = 23 confirmed cases) limits the statistical power of subgroup comparisons. Second, the study was conducted at a single diagnostic center, which may introduce selection bias toward clinically presented cases.

Third, treatment response, long-term follow-up, and recurrence rates were not evaluated, thereby limiting the assessment of therapeutic outcomes. Finally, molecular or cytogenetic confirmation of tumor clonality was not performed, although cytological and histopathological findings were highly consistent with classical CTVT morphology.

### Veterinary public health implications

The findings of this study highlight the importance of preventive veterinary public health measures. Given the strong association between CTVT and uncontrolled reproduction, effective control strategies should include the implementation of systematic sterilization programs, the reduction of stray and free-roaming dog populations, the enforcement of leash and ownership regulations, and public education on responsible pet ownership and disease transmission.

Such measures are essential for reducing transmission risk and controlling disease spread in urban environments such as Astana. Furthermore, the establishment of regional surveillance programs and routine screening of high-risk canine populations may facilitate earlier diagnosis and contribute to long-term disease control in Kazakhstan and other Central Asian countries.

## CONCLUSION

This study provides the first comprehensive clinicopathological characterization of CTVT in Kazakhstan and one of the few detailed reports from temperate Central Asia. Among 2,500 examined dogs, 23 cases of CTVT were identified, representing 5.4% of all diagnosed neoplasms. The disease occurred predominantly in male dogs, particularly those aged 1.5–5 years, and was most frequently observed in mixed-breed animals. Clinical presentation was characterized by friable, highly vascular genital masses accompanied by hemorrhagic discharge, whereas purulent complications were observed in more than half of the affected dogs. Extragenital localization was uncommon but confirmed CTVT’s capacity to develop beyond the reproductive tract.

Dogs with purulent complications exhibited more pronounced clinical and physiological disturbances, including tachycardia, tachypnea, leukocytosis, anemia, and alterations in biochemical parameters. Cytological examination consistently revealed characteristic round tumor cells with eccentrically positioned nuclei, coarse chromatin, prominent nucleoli, frequent mitotic figures, and the distinctive “string-of-pearls” cytoplasmic vacuolization pattern. Histopathological findings confirmed the cytological diagnosis, particularly in atypical and extragenital cases, emphasizing the diagnostic value of combining cytological and histopathological assessments.

A major strength of this study was the integration of epizootological, clinical, hematological, biochemical, cytological, histopathological, and microbiological evaluations within a single diagnostic framework. This multidisciplinary approach provides valuable baseline data for understanding the epidemiology and clinicopathological characteristics of CTVT in a previously underreported geographic region. However, the relatively small number of confirmed cases, the single-center design, the absence of molecular confirmation of tumor clonality, and the lack of treatment-response and long-term follow-up data should be considered when interpreting the findings.

The results highlight the importance of early diagnosis, routine cytological screening, and responsible reproductive management for reducing disease transmission. The implementation of sterilization programs, improved control of free-roaming dog populations, and public education on responsible pet ownership are essential components of disease prevention. Future multicenter studies incorporating molecular characterization, therapeutic evaluation, and long-term follow-up are warranted to further elucidate the epidemiology, biological behavior, and control of CTVT in Kazakhstan and throughout Central Asia. Overall, this study establishes an important regional reference for CTVT and provides evidence to improve diagnostic, surveillance, and disease management strategies in canine populations.

## DATA AVAILABILITY

The datasets generated and/or analyzed during the current study are not publicly available due to institutional restrictions, but are available from the corresponding author upon reasonable request.

## AUTHORS’ CONTRIBUTIONS

GKM and AP: Conceptualization, study design, clinical examination, sample collection, data acquisition, and edited the manuscript. KA: Clinical examination, sample collection, data acquisition, cytological analysis, histopathological analysis, and edited the manuscript. AA: Cytological analysis, histopathological analysis, statistical analysis, data interpretation, and drafted and revised the manuscript. DMK, AZ, YB, BB, BK, KKA, GO, and AK: Statistical analysis, data interpretation, and drafted and revised the manuscript. All authors have read and approved the final manuscript.
